# Habitual physical activity is associated with the maintenance of neutrophil migratory dynamics in healthy older adults

**DOI:** 10.1016/j.bbi.2016.02.024

**Published:** 2016-08

**Authors:** David B. Bartlett, Oliver Fox, Clare L. McNulty, Hannah L. Greenwood, Laura Murphy, Elizabeth Sapey, Martin Goodman, Nicola Crabtree, Cecilie Thøgersen-Ntoumani, James P. Fisher, Anton J.M. Wagenmakers, Janet M. Lord

**Affiliations:** a MRC-ARUK Centre for Musculoskeletal Ageing Research, Institute of Inflammation and Ageing, University of Birmingham, Birmingham B15 2TT, United Kingdom; b School of Sport, Exercise and Rehabilitation Sciences, University of Birmingham, Birmingham B15 2TT, United Kingdom; c Geriatric Medicine, University Hospitals Birmingham NHS Foundation Trust, Birmingham B15 2WB, United Kingdom; d NIHR/Wellcome Trust Clinical Research Facility, Queen Elizabeth Hospital, Birmingham B15 2WB, United Kingdom; e Research Institute for Sport & Exercise Sciences, Liverpool John Moores University, Liverpool L3 3AF, United Kingdom

**Keywords:** Neutrophil, Physical activity, Ageing, Innate immunity, Immunosenescence, Migration, Inflammation

## Abstract

**Background:**

Dysfunctional neutrophils with advanced age are a hallmark of immunosenescence. Reduced migration and bactericidal activity increase the risk of infection. It remains unclear why neutrophil dysfunction occurs with age. Physical activity and structured exercise have been suggested to improve immune function in the elderly. The aim of this study was to assess a comprehensive range of neutrophil functions and determine their association with habitual physical activity.

**Method:**

Physical activity levels were determined in 211 elderly (67 ± 5 years) individuals by 7-days of accelerometry wear. Twenty of the most physically active men and women were matched for age and gender to twenty of the least physically active individuals. Groups were compared for neutrophil migration, phagocytosis, oxidative burst, cell surface receptor expression, metabolic health parameters and systemic inflammation. Groups were also compared against ten young participants (23 ± 4 years).

**Results:**

The most active group completed over twice as many steps/day as the least active group (*p* < 0.001), had lower BMI’s (*p* = 0.007) and body fat percentages (*p* = 0.029). Neutrophils migrated towards IL-8 better in the most active group compared to the least active (*p* < 0.05) and was comparable to that of the young (*p* > 0.05). These differences remained after adjusting for BMI, body fat and plasma metabolic markers which were different between groups. Correlations revealed that steps/day, higher adiponectin and lower insulin were positively associated with migratory ability (*p* < 0.05). There was no difference in expression of the chemokine receptors CXCR1 or CXCR2 (*p* > 0.05 for both). CD11b was higher in the most active group compared to the least active (*p* = 0.048). No differences between activity groups or young controls were observed for neutrophil phagocytosis or oxidative burst in response to *Escherichia coli* (*p* > 0.05). The young group had lower concentrations of IL-6, IL-8, MCP-1, CRP, IL-10 and IL-13 (*p* < 0.05 for all) with no differences between the two older groups.

**Conclusion:**

These data suggest that impaired neutrophil migration, but not bactericidal function, in older adults may be, in part, the result of reduced physical activity. A 2-fold difference in physical activity is associated with better preserved neutrophil migratory dynamics in healthy older people. As a consequence increasing habitual physical activity may be beneficial for neutrophil mediated immunity.

## Highlights

• Physical activity associates with neutrophil migration in the elderly.
• Effects occur independently of inflammation or surface receptor expression.
• Adiponectin levels may positively influence neutrophil migration.

## 1 Introduction

Innate immunity is the first line of defence against invading microorganisms. With advancing age there is a chronic deterioration of immune function, termed immunosenescence, leading to increased susceptibility to infection (Gavazzi and Krause, 2002, Butcher et al., 2003). Neutrophils are the most abundant leukocyte in blood and the first immune cell to arrive at sites of bacterial infection, making them critical intermediaries in the effective resolution of infection. Neutrophil dysfunction is evident in healthy older adults and is characterised by reduced directional migration, phagocytosis, oxidative burst and neutrophil extracellular trap (NET) production (Butcher et al., 2001, Sapey et al., 2013, Hazeldine et al., 2014, Shaw et al., 2010). Reduced chemotaxis may be particularly detrimental as it will delay recruitment of neutrophils to sites of infection, but it may also contribute to heightened inflammation during infection and pro-inflammatory insults in older adults. This is because neutrophils release proteases such as neutrophil elastase to aid migration through tissue (Cepinskas et al., 1999), thus damaging healthy tissue and inducing inflammation (Sapey et al., 2013). Inefficient chemotaxis will increase this unintentional damage and inflammation. It remains unclear why neutrophil dysfunction occurs with ageing but it is likely that defects are intrinsic to the cellular maturation process as neutrophils exit the bone marrow as mature non-proliferating cells (Shaw et al., 2010, Ward et al., 2011, Fortin et al., 2008). Our own recent work has shown that signalling pathways central to chemotaxis, namely phosphoinositide 3 kinase (PI3K), is dysregulated in neutrophils from older adults and reduced chemotaxis can be corrected by selective inhibition of this pathway (Sapey et al., 2013). Identifying ways to prevent or reverse neutrophil immunosenescence is critical to improving immunity in older adults.

One of the features of ageing in humans is a reduced level of physical activity. Less than half of over 65 year olds in the UK meet national guidelines for habitual physical activity (House of Lords Select Committee, 2013). Importantly, a growing body of evidence suggests that regular participation in physical exercise can improve or maintain immune function in old age, potentially slowing the progression of immunosenescence (Walsh et al., 2011, Simpson et al., 2012). The question thus arises of whether a significant part of immunosenescence is the consequence of an inactive lifestyle, rather than being due to the intrinsic ageing process. Despite the extent of neutrophil dysfunction with age and its potential contribution to ill-health, there are relatively few studies assessing the effects of exercise on neutrophil function in the elderly. Yan and colleagues showed that phagocytosis was better maintained in physically active compared to sedentary older men (Yan et al., 2001). In overweight older women, 6-weeks of aerobic exercise training reduced circulating neutrophil numbers – a potentially beneficial anti-inflammatory effect (Michishita et al., 2010). In young participants, neutrophil chemotaxis and phagocytosis was improved following 2 months of moderate aerobic exercise training (Syu et al., 2012). These studies highlight the potential for regular exercise to positively modify neutrophil function and potentially lower the risk of infectious diseases in later life.

Understanding the effects of habitual physical activity on immune function in the elderly could lead to new models of exercise prescription for older adults. To date, no studies have assessed broad neutrophil functions in healthy older individuals with a range of physical activity levels. The aim of this study was thus to assess a comprehensive range of neutrophil functions known to be reduced with age and determine the association with habitual physical activity. We hypothesized that regular physical activity in later life would be associated with maintenance of neutrophil function. Specifically we assessed directional migration, cell receptor expression, phagocytosis of *Escherichia coli* and the oxidative burst response to *E. coli*.

## 2 Materials and methods

### 2.1 Participants and assessment of habitual physical activity

An assessment of physical activity and a broad range of physiological function were completed in 211 healthy older participants, 100 men (67 ± 5 years) and 111 women (66 ± 5 years), at the Wellcome Trust Clinical Research Facility (WTCRF), Birmingham, UK. For assessment of habitual activity levels participants wore a GT3X accelerometer (Actigraph, FL, USA) around the waist above the left leg for 7 days, except for during water based activities. Total steps per day were calculated using ActiLife Software v5 (Actigraph, FL, USA). Following 7 continuous days wearing an accelerometer the 20 most active (MA) and 20 least active (LA) participants were identified and asked to return to the WTCRF for an additional blood sampling. All participants were grouped (quintiles) by physical activity and then stratified for age and gender. The top ten MA males were then matched for age (±2 years) with ten LA males and the same was done for women. Upon recruitment to the study, participants again wore the accelerometer to ensure physical activity levels were similar to their first visit and completed the International Physical Activity Questionnaire (IPAQ). Details of participant age, physical activity levels, clinical and physiological characteristics are described in Table 1. Ten healthy young individuals (23 ± 4 years) were also recruited in order to compare neutrophil functions and serological status with age.

All participants were asked to abstain from physical exercise for 48 h before arriving at the clinic. Exclusion criteria was specified to ensure relatively good health and included: no general medical history in the previous 3 years: dementia, Parkinson’s, stroke, transient ischaemic attacks, liver disease, cancer, myocardial infarction, cardiomyopathy, valvular disease, angina pectoris, sudden chest pains, blood pressure >190/120 or tendencies to faint, asthma, chronic obstructive pulmonary disease, rheumatoid arthritis or osteoarthritis, use of corticosteroids, type 1 or 2 diabetes, leg pain during exertion, and consultation with GP for feeling unwell in the previous 10-days. Ethical approval was granted by the Black Country Research Ethics committee (10/H1202/77) and all participants gave written informed consent.

### 2.2 Clinical assessment

Clinical assessments were taken by trained research nurses in the WTCRF. Briefly height and weight were recorded and body composition was measured using dual-energy X-ray absorptiometry (GE Lunar iDXA, GE Healthcare, Madison, WI) using Encore Software v13.6, as previously described (Schousboe et al., 2013). Blood pressure (BP) and heart rate were taken after 15 min sitting, using an Omron M5-I automated BP machine (Omron Healthcare, Lake Forest, USA). An average of 3 BP readings taken over 5 min was taken as clinical BP (Omboni et al., 2007).

### 2.3 Functional testing

Each participant completed a short battery of physical functioning tests which are indicative of health and progression towards frailty in older people (Thrane et al., 2007, Nordin et al., 2008, Muir et al., 2008, Muir et al., 2010): Hand grip strength was determined by measuring both left and right hands using a hand held dynamometer (Takei TKK-5401, Takei Scientific Instruments, Japan) and the best scores used. Participants also completed the 6-metre timed-up and go (TUG) test, briefly, participants were timed to stand from sitting, walk 3-metres at their regular walking speed, turn around, walk back and sit down. The Berg Balance Scale (BBS), a measure of balance and postural stability, was also carried out in which participants were asked to complete 14 balance tasks and scores given. Scoring gives a risk of falling, with 41–56 a ‘low risk of falling’.

### 2.4 Blood sampling

Participants arrived at the clinic at 8am having fasted overnight and peripheral venous blood samples were taken into vacutainers containing either heparin, EDTA or a clotting agent for serum. Samples were then processed immediately for serum, plasma and immune cell isolation. Serum and plasma were separated from blood by centrifugation (3000*g* for 10 min), snap frozen in liquid nitrogen and stored at −80 °C until analysis.

### 2.5 Neutrophil isolation

Neutrophils were isolated from heparinised blood following dextran induced sedimentation and separation on a discontinuous Percoll gradient as previously described (Butcher et al., 2001). Neutrophil purity and viability was determined by Giemsa staining (Diff-Quik; Gentaur Europe, Brussels, Belgium) and trypan blue (Sigma Aldrich, Dorset, UK) exclusion, respectively. Neutrophil purity and viability were consistently ⩾96%. Neutrophils were then resuspended at 1 × 10^6^ ml^−1^ in RPMI 1640 medium (Sigma–Aldrich) containing 0.15% bovine serum albumin (Sigma–Aldrich).

### 2.6 Neutrophil migration

Neutrophil migratory dynamics were assessed using an Insall chamber (Weber Scientific International Ltd., Teddington, UK) as previously described (Sapey et al., 2013, Muinonen-Martin et al., 2010). Briefly, coverslips were coated with 7.5% culture tested bovine serum albumin (Sigma–Aldrich) and neutrophils adhered to this surface for 20 min at room temperature. The use of this bovine serum albumin has previously been shown to mimic the ligand for CD11b and CD18, ICAM-1 (Zhu et al., 2000). The coverslip was then inverted on the Insall chamber before addition of buffer (RPMI-1640) alone as a control or buffer containing 100 nM of interleukin (IL)-8 (R&D Systems; Abingdon, UK).

Neutrophil migration was monitored using a Zeiss Axiovert 100 inverted microscope fitted with a Fast Mono 12-bit QICAM digital camera. Time-lapse recordings and calculation of neutrophil migratory dynamics were performed as previously described (Sapey et al., 2013). Briefly, the Insall chamber allows the formation of stable chemoattractant gradients, with defined, consistent direction in the y direction for each experiment (Muinonen-Martin et al., 2010). Only distance travelled in the y direction over time was included in calculations of chemotaxis. Migration was assessed using 3 parameters: Average cell speed (μm/min) of movement towards a chemokine (e.g. IL-8, termed chemokinesis), average velocity (μm/min) of cells (termed chemotaxis) and accuracy of movement (termed chemotactic index). Chemotactic index is expressed in a comparative scale (CS) ranging from −1 to +1. Movement directly towards the chemoattractant is +1 whilst directly away is −1.

Recordings lasted 20 min per experiment, with 20 slides captured using Improvision OpenLab software. The Java software ImageJ (Wayne Rasband, NIH, Bethesda, MD) was used to analyse cell tracks. All analysis was carried out by a single analyst, blinded to subject group and cell conditions.

### 2.7 Neutrophil phagocytosis and oxidative burst

Phagocytosis and oxidative burst were assessed in whole blood using commercially available kits and manufacturers’ guidelines (BD Biosciences, Oxford, UK). Briefly, phagocytosis was assessed in heparin treated whole blood and incubated at 4 °C (control) or 37 °C (test) with opsonised FITC-labelled *E. coli*. Phagocytosis was halted by the addition of cold phosphate buffered saline (PBS) whilst cell surface bound FITC was quenched by addition of Trypan Blue solution. Unbound free bacteria were removed by washing in PBS and erythrocytes lysed and leukocytes fixed using 1% fix/lyse solution provided in the kit. Cell DNA was counterstained by addition of propidium iodide (PI) before flow cytometry analysis was performed.

Oxidative burst was assessed in heparin treated whole blood which was incubated at 37 °C with opsonised *E. coli* (test) or PBS (control). Solution containing dihyrdorodamine-123, which is converted to fluorescent rodamine-123 in the presence of reactive oxidants, was included for 10 min at 37 °C. Oxidative burst was halted by the addition of erythrocyte lysis/leukocyte fixation buffer before leukocyte DNA was stained and flow cytometry analysis performed.

Phagocytosis and oxidative burst quantitation was performed on a CyAn_ADP_™ 430 flow cytometer (Beckman-Coulter, High Wycombe, UK) equipped with 3 solid-state lasers. FITC and R-123 were detected in FL1 whilst PI was detected in FL2 using the Argon (405 nm) laser. 10,000 neutrophils were acquired for analysis. Following compensation of FL1 v FL2 phagocytic and oxidative burst were determined by the relative increase in percentage and median fluorescence intensity (MFI) in FL1 compared to negative controls. Data were analysed using Summit v4.3 (DAKO, Cambridgeshire, UK).

### 2.8 Measurement of surface receptor expression

Freshly isolated neutrophils (1 × 10^5^ ml^−1^) were stained with anti-CXCR1-FITC (eBioscience, Hatfield, UK, clone 8F1-1-4), anti-CXCR2-PE (eBioscience, clone 5E8-C7-F10), anti-CD16-APC (eBioscience, clone CB16), anti-CD16-FITC (BD Bioscience, clone 3G8), anti-CD11b-APC (BD Bioscience, clone ICRF44), anti-CD18-PE (BD Bioscience, clone 6.7) or their relevant concentration-matched isotype control for 30 min on ice in the dark. Following incubation, cells were washed in PBS/1% BSA and resuspended in PBS/1% BSA for analysis by flow cytometry. 10,000 CD16^+^ neutrophils were acquired for analysis on a CyAn_ADP_™ 430 flow cytometer and data analysed using Summit v4.3 software.

### 2.9 Serological and metabolic analysis

All measurements were made in duplicate using commercially available kits and manufacturers guidelines. Serum IL-1β, IL-4, IL-6, IL-8, IL-10, IL-13, IL-17, GMC-SF, TNFα and MCP-1 were simultaneously measured by multiplex luminometry (Bio-Rad, Hemel Hempstead, UK). Samples were analysed using a Bio-Plex Luminex^200^ platform equipped with a 635 nm red and 532 nm green laser using Bio-Plex Manager™ software. Detection of C-reactive protein (CRP) was by high sensitivity ELISA using a commercial kit (IBL International, Hamburg, Germany). Plasma adiponectin and leptin were assessed separately by solid phase sandwich ELISA (R&D Systems, Abingdon, UK). Plasma insulin levels were determined by sandwich ELISA (Life Technologies, Paisley, UK). Plasma glucose, cholesterol and triglycerides (Instrumentation Laboratories, Warrington, UK) were determined photometrically on an automated chemistry analyser (iLab 650, Instrumentation Laboratories, Warrington, UK).

#### 2.10 Statistical analysis

All analyses were conducted using PASW version 18.0 (Chicago, IL, USA) and all data presented as mean ± standard deviation (SD) unless otherwise stated. Normality was assessed using Kolmogrov-Smirnov analysis; natural log transformation of distributed variables violating normality was completed. Analysis of covariance (ANCOVA) with Bonferroni correction and independent sample T-tests were used to determine differences between the groups. Covariates were included in the analyses if they were different between the most and least active elderly groups. Pearson correlation analysis was conducted to determine relationships between migratory dynamics and steps/day as well as metabolic variables. The homeostasis model assessment of insulin resistance (HOMA-IR) was calculated as described previously using the HOMA2-IR online calculator (The Oxford Center for Diabetes, 2014, Wallace et al., 2004). As previously described, all experiments were preceded by studies of intra-subject and inter-subject variance to allow appropriate power calculations to be performed (Sapey et al., 2013). Statistical significance was accepted as *p* < 0.05.

## 3 Results

### 3.1 Participant characteristics

The physical and serological characteristics of the participants are shown in Table 1. Physical activity levels ranged from 1518 to 19,161 steps/day and as is typical with older people a low percentage, only 13%, completed >10,000 steps/day, causing a left-shift in distribution. There were no differences between the first and second wearing of the accelerometers and both were highly correlated, (r(38) > 0.784; *p* < 0.001 for all), therefore data presented here are from the second wear time only. The most active (MA) group was over twice as active for steps/day as the least active (LA) group (p < 0.001). These data highlighted that not only were the MA group walking more, but were less sedentary. Results from self-perceived activity reports (IPAQ) were similar to accelerometer data with approximately a twofold difference between the two groups (*p* = 0.026).

Although attempts were made to match the groups for as many potential confounding variables as possible, inevitably a number of differences were apparent. In particular, body mass index (BMI; *p* = 0.007) and body fat percentage (*p* = 0.029) were higher in the LA group which likely accounted for a number of metabolic differences. Fasting glucose (*p* = 0.010), insulin (*p* = 0.004) and leptin (*p* = 0.036) were all higher whilst adiponectin (*p* = 0.047) was lower, in the LA group. Importantly, none of these measures were clinically abnormal and other measures of health including blood pressure, resting heart rate, triglycerides, total cholesterol, grip strength, balance, walk speed, statin medication, and smoking were similar between groups (*p* > 0.05 for all). There were also no differences in total immune cell counts for neutrophils, monocytes or lymphocytes between the two groups (Table 1). Taken together, these data show that our two populations were relatively healthy and we can be confident that any group differences are likely driven by differences in physical activity levels rather than morbidity.

### 3.2 Neutrophil migration and physical activity

**Fig. 1:**
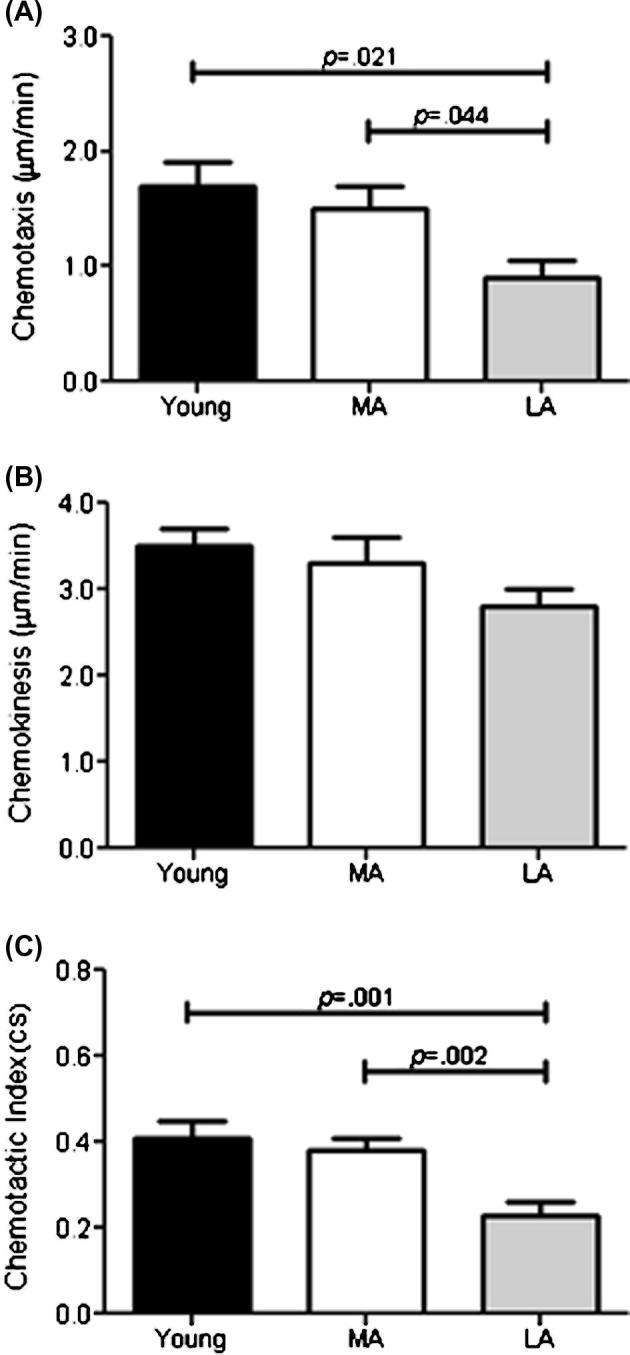
Differences for neutrophil chemotaxis (A), chemokinesis (B) and chemotactic index (C) for cells isolated from Young (n = 10), Most active (MA) and Least active (LA) older participants (n = 20 each). Data are mean ± SEM.

Efficient neutrophil migration is a critical step in the resolution of infection and has recently been shown to be impaired with advancing age (Sapey et al., 2013). In agreement with published data, isolated neutrophils from young participants migrated towards the chemokine IL-8 with a greater directional speed [chemotaxis (*p* = 0.038)] and an overall greater chemotactic accuracy [chemotactic index (*p* = 0.020)], but not overall speed [chemokinesis (*p* = 0.098)], than older participants. When split into their respective activity groups, the LA group had significantly reduced neutrophil chemotaxis (Fig. 1A) compared to both the MA (*p* = 0.044) and young participants (*p* = 0.021) and there were still no differences between groups for chemokinesis (Fig. 1B). Chemotactic index for the LA group (Fig. 1C) was also reduced compared to the MA group (*p* = 0.002) and young participants (*p* = 0.001).

Correlation analysis revealed that increased steps/day were associated with increased neutrophil chemotactic index (r(38) = 0.689, *p* = 0.001) but not chemotaxis or chemokinesis (both *p* > 0.05). These data suggest being physically active in later life could maintain neutrophil migratory dynamics, specifically accuracy and velocity, and that the amount of physical activity is related to the degree of migratory benefit.

### 3.3 Neutrophil migration and a relationship with metabolic phenotype

**Fig. 2:**
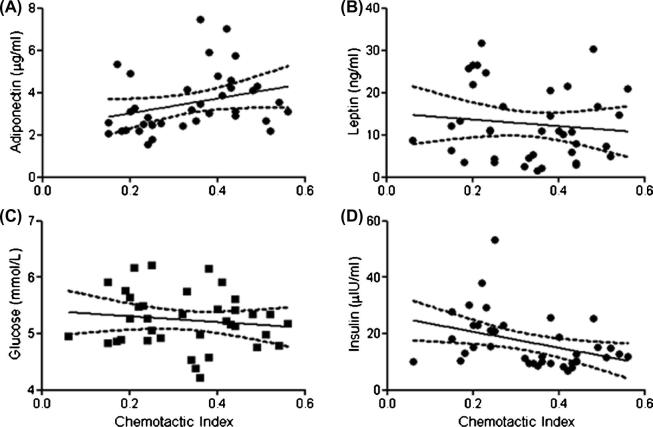
Correlation analyses plus 95% confidence intervals of metabolic variables for all older participants (n = 40) and their relationship with Chemotactic Index for Adiponectin (A), Leptin (B), Glucose (C), and Insulin (D).

In order to determine if the relationship between neutrophil migration and physical activity might be mediated by differences in metabolic parameters between the LA and MA groups, data was analysed to include BMI, body fat percentage, adiponectin, leptin, glucose and insulin concentrations as covariates. Inclusion of these variables did not statistically alter our findings for any individual migratory parameters, chemokinesis, chemotaxis or chemotactic index (Supplementary Table 1). Correlation analysis did however reveal that certain metabolic markers were associated with the overall chemotactic index. Fig. 2A shows that adiponectin (r(38) = 0.515, *p* = 0.05) and insulin (Fig. 2D: r(38) = −0.617, *p* = 0.01) were associated with chemotactic index, whilst leptin (Fig. 2B: r(38) = −0.359, *p* = 0.22) and glucose (Fig. 2C: r(38) = −0.327, *p* = 0.263) were not. These data suggest that exposure to raised adiponectin and reduced insulin may play a role in mediating the effects of exercise on neutrophil migration.

### 3.4 Phagocytosis and oxidative burst and relation to physical activity

**Fig. 3:**
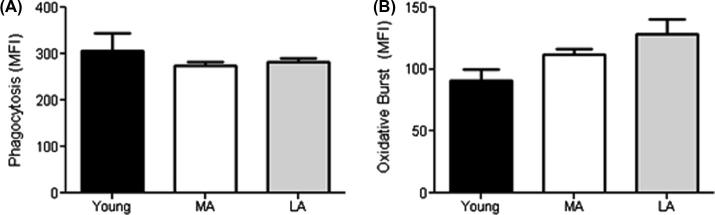
Differences between the Young, Most (MA) and Least Active (LA) older participants for neutrophil uptake of FITC-labelled *E. coli* (A) and the oxidative burst against ingested *E. coli* (B). Data are mean ± SEM.

A primary mechanism of bacterial clearance by neutrophils is ingestion through phagocytosis and subsequent killing in the phagolysosome through exposure to reactive oxygen species such as superoxide anions. All neutrophils ingested bacteria and produced an oxidative burst. Phagocytosis of opsonised FITC-labelled *E. coli* showed no differences between young, LA or MA groups (Fig. 3A) and the oxidative burst also did not differ between groups (Fig. 3B).

### 3.5 Neutrophil receptor expression

To understand whether the differences in migration were due to the detection of the chemokine IL-8, or the adhesion to the coverslip chemokine and adhesion receptor expression was assessed on isolated neutrophils (Table 2). All neutrophils expressed the receptors measured. No differences were observed between groups for the IL-8 receptors CXCR1 and CXCR2, or the adhesion molecule CD18. There was a small group effect for the adhesion molecule CD11b [F_(2, 39)_ = 3.64, *p* = 0.036, η^2^ = 0.168] which was due to lower expression on the LA neutrophils compared to the MA (*p* = 0.048) and young (*p* = 0.041) groups. CD11b was included as a covariate in the analysis of Chemotactic Index but did not alter our findings. Our data suggest that differences in neutrophil migration are not the result of chemokine receptor expression.

### 3.6 Systemic inflammation and neutrophil migratory dynamics

Exposure to inflammatory mediators is known to influence the function of neutrophils including migration. In order to determine whether systemic inflammation could have contributed to migratory differences levels of pro-inflammatory cytokines, acute phase proteins and anti-inflammatory cytokines were assessed. Table 3 reveals the young subjects had lower levels of circulating IL-6 (*p* = 0.011), IL-8 (*p* = 0.049), MCP-1 (*p* = 0.002), CRP (*p* = 0.039), IL-10 (*p* = 0.004) and IL-13 (*p* = 0.033) than the combined older participants. No differences between activity groups for the LA and MA groups were evident.

## 4 Discussion

With a growing body of evidence to suggest that physical activity in later life reduces the risk of age related diseases it is imperative to understand which specific aspects of physiology benefit from a healthy lifestyle (Brandt and Pedersen, 2010, Pedersen, 2011). The immune system characteristically becomes dysregulated with age leading to a severe functional loss and exposing older individuals to increased risk of infection and disease (Pawelec et al., 2005, Panda et al., 2009). The beneficial effects and impact of physical activity on the immune system, especially adaptive immunity, have been extensively studied in young healthy individuals (LaVoy et al., 2013, Simpson et al., 2010, Spielmann et al., 2011). However few studies have specifically focused on the neutrophil in older adults and the effects of habitual physical activity in later life. This study is the first to compare neutrophil phagocytosis, superoxide production and detailed migratory dynamics towards the chemokine IL-8 in habitually physically active and less active healthy older individuals. We show for the first time that a 2-fold difference in physical activity is associated with better preserved neutrophil migratory dynamics in healthy older people.

Neutrophil migration is a key component for the resolution of infection and control of inflammation. Along with age-associated reductions in bacterial clearance, migratory dysfunction can lead to increased morbidity and mortality from bacterial infections (Liang and Mackowiak, 2007). Defective migration results in poor resolution of inflammation and wound repair in aged mice (Brubaker et al., 2013). Additionally, if the neutrophil does not migrate accurately between tissues, collateral damage is augmented by elevated proteinase release from the cell, enhancing the inflammatory milieu and potentially contributing to the increased infection associated frailty (Liou and Campbell, 1996, Pham, 2006, Fleming and Elliot, 2005). Recent work from our group showed that neutrophils from older adults migrated with a similar speed but reduced velocity and accuracy when compared to younger participants (Sapey et al., 2013). Our findings here show that velocity and accuracy were similarly reduced in our older cohort compared to young subjects, but we now add that the reduced habitual activity of our elders is associated with this loss of migratory accuracy.

A number of studies have highlighted the beneficial effects of physical exercise on immune function in the young and to a lesser extent the old. However, few studies have assessed whether neutrophil migration is associated with physical activity. Neutrophil migration was improved following six months of moderate exercise training in hypertensive older women (65 ± 5 years) to a similar level of migration as that in age-matched healthy women (Fuente et al., 2005). Furthermore, migration was also improved 2-fold in young sedentary individuals following a 2 month moderate intensity cycling exercise program (Syu et al., 2012). Interestingly, in both these studies following a period of no-training or de-training neutrophil migration reverted back to levels prior to commencing training. These data suggest that efficient neutrophil migration is not only improved by physical exercise but is highly plastic and fundamentally dependent on it. In support of a physical activity effect on neutrophil function, we show that just a 2-fold difference in habitual physical activity levels was sufficient to be associated with increased neutrophil chemotaxis and chemotactic index. Moreover, none of our participants were actively engaged in structured exercise training as stated in their IPAQ’s, thus higher habitual physical activity levels resulted in significantly better migration.

To investigate the potential mechanisms we assessed whether cell surface receptor expression related to migratory dynamics was altered with physical activity levels. The two main neutrophil receptors for IL-8 are the G-coupled protein receptors CXCR1 and CXCR2, which signal through a range of pathways, with the PI3K/AKT and Rac GTPase pathways central to chemotaxis and migratory accuracy (King and Insall, 2009). CXCR2 has been shown to increase in muscle fibres and vascular endothelium following acute exercise, likely stimulating angiogenesis in the working tissue (Frydelund-Larsen et al., 2007). However we found that physical activity levels did not correlate with CXCR1 or CXCR2 expression on neutrophils. Our group recently showed that neither CXCR1 nor CXCR2 were altered with age and instead migratory dysfunction was associated with constitutive activation of PI3K, which if inhibited pharmacologically led to improved chemotaxis (Sapey et al., 2013). Whether higher habitual physical activity can reduce the constitutive PI3K activity seen in neutrophils with age was not determined here, but could offer an explanation for the association observed. Thus migratory defects due to physical inactivity are likely intrinsic to downstream signalling differences and possibly through modification of PI3K activity.

Of the adhesion receptors measured only CD11b was different between active and inactive elders, with around a 2-fold reduced expression in less active elders. CD11b is an α-integrin which forms a complex with the β2-integrin CD18 to form the adhesion complex MAC-1. Together this complex is important for firm adhesion to vascular endothelial walls where it binds to ICAM-1 before transmigrating into the tissue. In our model, the albumin used to coat the coverslips mimics ICAM-1 (Zhu et al., 2000). Basal neutrophil expression of CD11b has been shown to be unaltered with age (Butcher et al., 2001, Murciano et al., 2008). Priming neutrophils with TNFα up-regulates expression of MAC-1 and promotes migratory function (Montecucco et al., 2008). Interestingly, TNFα mediated MAC-1 expression is dependent on PI3K/Akt and p38 MAPK activity (Montecucco et al., 2008). Therefore it may be that the increased expression of CD11b on neutrophils from active elders confers an efficient migratory phenotype through a functional PI3K/Akt signalling pathway similar to younger individuals. It remains unclear whether an increased basal expression of CD11b results in improved neutrophil migration in elders who are physically active. CD11b but not CD18 neutrophil expression has been shown to be upregulated following acute bouts of exercise and exercise training (Jordan et al., 1999). Therefore it may be that regular physical activity promotes surface expression of CD11b as a surveillance mechanism readying neutrophils to migrate to sites of infection.

One unexpected result was the lack of a difference in phagocytosis or ROS production between any of the groups. We have previously shown age-associated reductions in phagocytosis and others have reported reductions in ROS production in response to *Staphylococcus Aureus* but not to *E. coli*, findings for these parameters are thus variable in the literature (Butcher et al., 2001, Wenisch et al., 2000). The data for habitual physical activity do suggest however that this does not modify these aspects of neutrophil function. One difference between these bactericidal assays and neutrophil migration is the stimulus for migration was a single chemokine (IL-8), whereas ingestion of the bacteria and ROS generation is effected by a range of signalling pathways and receptors which may not be sensitive to physical activity.

Neutrophils exposed to chronic inflammatory stimulants like TNFα and IL-1β, such as occurs in rheumatoid arthritis, have altered function (Wright et al., 2010). This includes extended lifespan and increased inflammatory cytokine and chemokine production augmenting the inflammatory milieu (Raza et al., 2006, Cross et al., 2004, Parsonage et al., 2008). Migratory activity is also altered in these conditions with cells becoming more adherent (Álvarez-Rodríguez et al., 2013, Aglas et al., 1998, Paino et al., 2011, Stockley et al., 2013). Increased systemic inflammation with age, inflammaging, has been well documented and we observed higher systemic inflammation in the older group as a whole compared to the young. However no differences were observed between high and low physical activity groups. This is in contrast to a number of studies suggesting that exercise can reduce systemic inflammation and that physical inactivity is associated with elevated inflammation (Pischon et al., 2003, Colbert et al., 2004, Nicklas et al., 2008). This might be reflective of our recruitment criteria and the health of our participants. In addition it is possible that our sample size lacked power to detect a difference in the older group with activity level. Thus, the migratory differences observed between activity groups and the young were likely not a consequence of chronic exposure to systemic inflammation.

Regular physical activity is known to positively alter the metabolism of individuals and reduce the risk for type-2 diabetes and associated cardiovascular disorders (Gleeson et al., 2011). Indeed, we observed a significantly lower measure of insulin resistance and diabetes risk (HOMA-IR) in the MA group (*p* = 0.004). Lower circulating insulin and fasting glucose levels coupled with reduced body fat and adipocyte related inflammation are a common feature of physically active individuals. Additionally, insulin signals through the PI3K/AKT pathway also and may account for altered PI3K activation in the elderly with increased circulating insulin concentrations (Schulman et al., 2007). Recently neutrophils were shown to facilitate insulin resistance in obese mice, highlighting a specific connection between innate immune function and metabolism (Talukdar et al., 2012). Adipokines such as adiponectin and leptin are important regulators of energy metabolism and their ratio is indicative of insulin sensitivity (Inoue et al., 2005, Inoue et al., 2006). Additionally both have been suggested to modify neutrophil function (Caldefie-Chezet et al., 2001, Caldefie-Chezet et al., 2003, Lang and Ratke, 2009). In this study we found no association between serum leptin and chemotactic index. Adiponectin on the other hand plays an important role in the regulation of energy metabolism via AMPK signalling and has inherent anti-inflammatory effects. Here adiponectin levels were positively associated with chemotactic index. Our group showed adiponectin delayed spontaneous neutrophil apoptosis whilst others have shown reduced IL-8 production in obese individuals associated with reduced serum adiponectin (Rossi and Lord, 2013, Trellakis et al., 2012). Recently, adiponectin was associated with improved neutrophil migration towards acute kidney injury in mice (Jin et al., 2013). A recent study suggested that the addition of 20-weeks aerobic exercise to dieting in overweight postmenopausal women resulted in increased adiponectin concentrations (Wang et al., 2015). Together these findings suggest a positive role for adiponectin on neutrophil function and migration. Further work is required to understand metabolism in neutrophils and the role exercise plays.

Our study is not without limitations. We did not measure body composition, physical activity levels or physical functioning in our young cohort. Our data suggest that neutrophil dysfunction, specifically chemotactic index, may be a consequence of physical inactivity. This would have been further supported had the activity levels of the young cohort matched the most active elderly. Furthermore we did not consider dietary differences between groups. It is clear that different compositions (carbohydrate, protein, fats) of diet can have effects on metabolism and therefore potentially cell function. However, we measured a number of inflammatory and endocrine markers which are associated with poor diet and increased adiposity and determined they did not have a direct effect on neutrophil migration. Future studies assessing immunological differences between young and old individuals should consider physical activity and dietary differences. Finally we have previously observed that PI3K is constitutively activated in elderly individuals and blocking PI3K in these cells restores migratory dynamics. Future studies should assess PI3K expression associated with physical activity and determine where the signalling defect occurs.

In conclusion our study suggests impaired neutrophil migration, but not bactericidal function, in older adults may be in part the result of physical inactivity. It is unlikely that these effects are driven by systemic inflammation or reduced surface chemokine receptor expression but a positive correlation with adiponectin levels suggests an influence of this metabolic hormone. Increasing habitual physical activity levels in elderly individuals may therefore be beneficial for neutrophil mediated immunity.

## Conflicts of Interest

All authors declare they have no conflicts of interest.

## Figures and Tables

**Table 1:** Characteristics of the forty older participants assessed.

	Elderly activity group	
	Most active (MA)	Least active (LA)
Gender (M/F)	10/10	10/10
Age (years)	67.4 ± 5.0	66.6 ± 4.9

*Physical Activity*		
Steps (per day)	10370 ± 3221	4746 ± 1351^∗∗∗^
IPAQ (AU)	29.4 ± 16.9	13.4 ± 11.3^∗^

*Physical Functioning*		
Grip Strength (kg)	30 ± 9	32 ± 9
Berg Balance Score (AU)	55.2 ± 1.4	55.3 ± 1.1
Timed-up-and-go (seconds)	8.1 ± 1.5	8.4 ± 1.4

*Clinical*		
BMI (kg/m^2^)	23.5 ± 3.1	26.7 ± 3.9^∗∗^
Body Fat (%)	26.2 ± 7.9	31.5 ± 6.7^∗^
Blood Pressure		
Systolic (mmHg)	128 ± 16	127 ± 13
Diastolic (mmHg)	77 ± 6	77 ± 7
Resting Heart Rate (bpm)	65 ± 9	64 ± 8
Current Smoker (Y/N)	0/20	2/18
Use of Statins (Y/N)	1/19	5/15

*Physiological*		
White Blood Cells		
Neutrophils (10^9^/L)	3.08 ± 0.8	3.34 ± 0.8
Monocytes (10^9^/L)	0.27 ± 0.08	0.32 ± 0.12
Lymphocytes (10^9^/L)	1.28 ± 0.28	2.00 ± 1.64
Fasting Glucose (mmol/L)	5.0 ± 0.4	5.4 ± 0.5^∗^
Fasting Insulin (μIU/ml)	12.3 ± 4.1	20.9 ± 11.5^∗∗^
HOMA-IR (AU)	2.8 ± 1.1	5.1 ± 3.2^∗∗^
Triglycerides (mmol/L)	1.14 ± 0.56	1.16 ± 0.62
Cholesterol [statin^+^ (mmol/L)]	5.80	4.15 ± 0.47
Cholesterol [statin^−^ (mmol/L)]	5.75 ± 0.97	5.04 ± 1.34
Leptin (ng/ml)	9.9 ± 7.8	15.7 ± 8.7^∗^
Adiponectin (μg/ml)	4.2 ± 2.0	3.1 ± 1.1^∗^
Leptin: Adiponectin Ratio	2.5 ± 1.9	5.5 ± 3.7^∗∗^

IPAQ (International Physical Activity Questionnaire); AU (Arbitrary Units); BMI (Body Mass Index); HOMA-IR (Homeostasis Model Assessment for Insulin Resistance); Statin^+^ (Prescribed statins); Statin^-^ (Not prescribed statins). Data are mean ± SD unless indicated otherwise. ^*^p < 0.05, ^**^p < 0.01, ^***^p < 0.001 compared to MA.

**Table 2:** Surface receptor expression (MFI) of key mediators of neutrophil adhesion and migration between young (n = 10) and older participants (n = 40). Data are mean ± SEM.

	Young	Old		
All	MA	LA
*Chemokine Recognition*				
CXCR1	55.1 ± 2.8	58.3 ± 1.5	57.3 ± 2.5	59.3 ± 1.6
CXCR2	61.9 ± 12.2	47.4 ± 4.2	53.5 ± 6.3	41.6 ± 5.4

*Adhesion Molecules*				
CD11b	26.6 ± 4.5	19.8 ± 2.3	25.2 ± 4.2	14.6 ± 1.6^∗#^
CD18	39.3 ± 5.3	40.2 ± 3.7	45.6 ± 5.9	35.1 ± 4.3

CXCR (CXC Chemokine Receptor); CD (Cluster of Differentiation). ^*^p < 0.05 and ^#^*p* < 0.05 compared to MA and young respectively

**Table 3:** Concentrations of pro- and anti-inflammatory mediators between the young (n = 10) and older (n = 40) participants. Values are pg/ml unless stated otherwise and data are mean ± SEM.

	Young	Old		
All	MA (n = 20)	LA (n = 20)
*Pro-Inflammatory*				
IL-1β (pg/ml)^a^	0.21 ± 0.05	0.33 ± 0.04	0.31 ± 0.05	0.35 ± 0.07
IL-6 (pg/ml)^a^	1.36 ± 0.92	5.11 ± 1.49^#^	5.71 ± 2.27	4.46 ± 1.99
IL-8 (pg/ml)	5.63 ± 0.58	6.79 ± 0.30^#^	6.49 ± 0.32	7.11 ± 0.51
IL-17 (pg/ml)^a^	10.09 ± 10.09	16.01 ± 6.55	17.40 ± 11.33	14.48 ± 6.13
TNFα (pg/ml)^a^	0.23 ± 0.11	3.99 ± 2.47	5.36 ± 4.40	2.48 ± 1.96
CRP (mg/L)^a^	1.30 ± 0.81	2.13 ± 0.45^#^	2.46 ± 0.81	1.77 ± 0.29
GM-CSF (pg/ml)^a^	ND	2.13 ± 1.52	2.44 ± 2.44	1.79 ± 1.79
MCP-1 (pg/ml)	27.63 ± 2.44	43.84 ± 2.57^##^	43.31 ± 3.84^#^	44.42 ± 3.44^#^

*Anti-Inflammatory*				
IL-4 (pg/ml)^a^	ND	0.01 ± 0.01	0.02 ± 0.01	0.01 ± 0.01
IL-10 (pg/ml)^a^	7.12 ± 3.12	19.34 ± 3.45^##^	17.33 ± 4.67	21.56 ± 5.19^#^
IL-13 (pg/ml)	4.13 ± 1.20	7.15 ± 0.71^#^	6.29 ± 0.87	8.10 ± 1.14

ND (not-detectable); IL (Interleukin); TNFα (tumour necrosis factor-α); CRP (C-Reactive Protein); GM-CSF (Granulocyte/Monocyte Colony Stimulating Factor); MCP-1 (Monocyte Chemoattractant Protein-1). ^a^Not normally distributed and log-transformed. ^#^*p* < 0.05 and ^##^*p* < 0.01 compared to young group.
