# Supplementary material for: Habitual physical activity is associated with the maintenance of neutrophil migratory dynamics in healthy older adults
Source: Brain Behav Immun. 2016 Aug;56:12–20. doi: 10.1016/j.bbi.2016.02.024 (PMC4929133; doi:10.1016/j.bbi.2016.02.024)
Supplement: Supplementary data [file mmc1.docx]

#### **Supplementary Table 1**: ANCOVA results for differences in migration between least and most active elderly individuals with metabolic parameters included as covariates

|  | Chemotaxis | | Chemokinesis | | | Chemotactic Index | | | |
| --- | --- | --- | --- | --- | --- | --- | --- | --- | --- |
|  | F | *p* | | F | *p* | | F | *p* |  |
| Covariate |  |  | |  |  | |  |  |  |
| BMI (kg/m^2^) | 4.1 | 0.051 | | 5.7 | 0.022 | | 11.3 | 0.002 |  |
| Body Fat (%) | 2.9 | 0.097 | | 7.2 | 0.011 | | 11.1 | 0.002 |  |
| Adiponectin (μg/ml) | 2.0 | 0.166 | | 4.8 | 0.036 | | 13.1 | 0.001 |  |
| Leptin (ng/ml) | 3.3 | 0.076 | | 6.5 | 0.015 | | 11.1 | 0.002 |  |
| Glucose (mmol/L) | 2.8 | 0.147 | | 6.5 | 0.015 | | 12.1 | 0.001 |  |
| Insulin (μIU/ml) | 0.5 | 0.503 | | 3.4 | 0.048 | | 9.3 | 0.004 |  |

BMI (Body Mass Index)
